# Impacts of Fire-fighting Chemicals on Native Fauna and Ecosystems in Australia: Identification of Key Knowledge Gaps and Research Priorities

**DOI:** 10.1007/s00267-025-02143-z

**Published:** 2025-03-24

**Authors:** Clare Morrison, Laura F. Grogan, Nick Clemann, Chantal Lanctôt

**Affiliations:** 1 Centre for Planetary Health and Food Security and School of Environment and Science, Griffith University, Southport, QLD 4222 Australia; 2 Wildlife Conservation & Science, Zoos Victoria, Parkville, VIC Australia; 3 Australian Rivers Institute and School of Environment and Science, Griffith University, Southport, QLD 4222 Australia; 4 Present Address: School of the Environment, University of Queensland, Brisbane, QLD 4072 Australia

**Keywords:** Fire-fighting chemicals, Bushfire, Wildfire, Australia

## Abstract

Increasing global wildfire frequency and intensity due to climate change has led to increasing use of fire-fighting chemicals (FFCs). While there is information relating to the environmental impacts of these FFCs in some regions, to date, there is little information on the impacts of FFCs on native fauna species and ecosystems in an extremely fire-prone country, Australia. We elicited input from a national-level interdisciplinary cohort of experts in fire research and management to identify fundamental gaps in knowledge and research priorities in the use and ecological impacts of FFCs in Australia. We used an anonymized online survey consisting of 21 close-ended, Likert-style and open-ended questions to collect expert opinion on the impacts and management of fire in Australia, focusing on the key knowledge gaps and research priorities relating to FFC use. Knowledge gaps and research priorities were identified in the areas of (1) the different types of FFCs recommended for use in Australia, (2) short and long-term effects of FFCs on fauna species and ecosystems, particularly in aquatic systems, and (3) interactions between FFCs and other environmental stressors. Addressing these knowledge gaps and research priorities will provide scientific-based evidence and recommendations for FFC use to inform future fire management practice and policy in Australia and can guide similar approaches in other countries requiring extensive FFC use for wildfire management.

## Introduction

Rising global temperatures and increased extreme weather have led to a dramatic increase in the number of extensive wildfires burning across the globe each year. Over the past 20 years, countries including Greece, Italy, Spain, Portugal, Russia, Canada, the United States and Australia have been seeing repeated large-scale burning resulting in >110 Mha of forest loss globally due to wildfire between 2001 and 2019 (Bousfield et al. 2023; MacCarthy et al. 2023; Tyukavina et al. 2022). During the summer of 2019–2020, over 15,000 fires burned across Australia, resulting in a combined impact area of up to 19 Mha (Filkov et al. 2020). The fires started during winter of Australia’s hottest and driest year on record (with the highest recorded Forest Fire Danger Index), and much of the land that burnt had already been impacted by severe drought (RCNNDA 2020). Almost three billion native vertebrates are estimated to have perished within the burned habitats, comprising more than 143 million mammals, 2.46 billion reptiles, 180 million birds, and 51 million frogs (World Wildlife Fund 2020). While Australia is a fire-prone country, with several major fire seasons and events on record (Rumpff et al. 2023), due to the unprecedented extent, duration and impact of these fires, the summer of 2019–2020 is now referred to as the ‘Black Summer’.

To combat multiple large fires during the Black Summer, ground and aerial fire fighters used several fire-fighting strategies, including large-scale aerial water bombing. However, the extreme fire-related weather made it challenging to fly accurate fire paths or get close to fire fronts on the ground, resulting in a shift from direct attacks on the fire fronts to widespread fire retardant drops on the green side of firebreaks (Wilson 2021). While there are regulations and recommendations for deploying fire-fighting chemicals (FFCs) in Australia, during wildfires, the priority is always saving lives, followed by infrastructure, with biodiversity usually the last priority (Rumpff et al. 2023). In extreme circumstances, such as the Black Summer fires, FFCs are sometimes dropped in protected areas not normally exposed to anthropogenic contaminants, including drinking water catchments and habitats of threatened species.

Using FFCs to assist with wildfire control and planned burns is a complex issue. While different FFCs and compounds provide a range of opportunities to improve fire-fighting efficiency and efficacy, the risks associated with using these products must be carefully considered and managed by fire and land management agencies (AFAC 2016). While most FFCs currently approved for use in Australia are purported to be environmentally safe compared to earlier FFC formulations, especially those containing PFAS (Squadrone et al. 2015), there is little information publicly available on their effects on Australian wildlife and ecosystems (see review by Gould et al. 2000). In many cases, in the absence of Australian standards relating to the environmental impacts of FFCs, it is recommended that only FFCs that have been approved by the United States Department of Agriculture (USDA) be used (AFAC 2016). This lack of context-specific information on the environmental impacts of FFCs makes it difficult to provide science-based recommendations for FFC use and policy in Australia. Given the predicted increase in wildfire risk due to climate change (Dowdy et al. 2019), the use of FFCs to manage and control more frequent and intense fires is also expected to increase. In fact, NSW Rural Fire Services reportedly used ten times more retardant (approximately 24 million liters) during the Black Summer bushfires compared to the previous years (NSW Government 2020). Rigorous and relevant science specific to the Australian context is, therefore, necessary to inform policy decisions and applications of FFCs (Sutherland et al. 2011).

Our study aimed to (1) collect and collate expert opinion on the impacts of FFCs on native fauna species and ecosystems, and (2) identify key knowledge gaps and research priorities in relation to FFC use for the management of wildfires in Australia. The information derived from this study can benefit three main groups of stakeholders. Policy makers, wildfire managers and practitioners in government, private and non-profit organizations can guide a research strategy to meet their information needs for policy development and implementation concerning FFC use. Research funding bodies can use the information to prioritize FFC research themes and programs jointly identified by researchers and end-users. Finally, researchers can better design and apply their FFC research programs to address the priority questions and issues highlighted by policy makers, managers, practitioners, and other end-users. While the study is focused on the Australian context, the methods used and information collected will be useful to stakeholder groups working with FFCs to manage wildfires in other countries.

## Methods

### Online Survey Design

Following the multi-step approach summarized below, we designed an anonymous online questionnaire to collect expert opinion on wildfire fighting chemical (FFC) use and impacts on Australian fauna species and ecosystems.

First, we reviewed the literature on FFC use and impacts on native fauna species and ecosystems in Australia to identify what research had been done, where, on what species/ecosystems, with which FFCs, and where knowledge gaps remained. We used a combination of search terms in Scopus and Web of Science to identify and collate peer-reviewed articles published in any year (see Supplementary Material for more details). We then developed the initial questionnaire using information, concepts and terminology found in the literature. The questionnaire design followed best-practice recommendations, including writing questions rather than statements, avoiding negatively worded questions, using an appropriate number of responses to questions, including a mix of question types, and ensuring the survey was an appropriate length (Artino et al. 2014). We did not provide the results of the literature review in the survey as we did not want to influence the experts’ responses.

The questionnaire was constructed as an online survey in Google Forms and pilot-tested with nine experts from academia, government agencies and fire-fighting organizations in February 2021. The trial survey aimed to determine (i) whether all questions were relevant, (ii) if key questions or responses had been omitted, (iii) whether the order of the questions/sections made sense, (iv) whether the questions and potential responses were clear, (v) whether the language and terminology used were appropriate, and (vi) the length of time needed to complete the questionnaire. The survey was modified using this feedback to improve clarity, wording and structure.

The final survey was completed in May 2021 (see Supplementary Material for survey) and comprised 21 close-ended, Likert-style and open-ended questions divided into five main sections: (1) Demographic information of participants, (2) Knowledge and experience with fire management and impacts of fire, (3) Knowledge gaps in relation to fire management and biodiversity, (4) Knowledge of FFCs – impacts, vulnerable taxonomic groups, interactions with other stressors, and (5) Organizational information needs concerning FFCs for fire management.

### Survey Distribution

We distributed the survey to experts from academia, government agencies, NGOs, fire services and other relevant organizations through targeted emails to known experts in the field, announcements through academic research centers, government agency networks, fire services personnel, professional environmental body mailing lists and newsletters, among others. Participants were encouraged to pass the survey details to other experts we may not have reached through our networks, i.e., the snowball technique. The survey was available from 1 June 2021 until 30 September 2021.

### Survey Anonymity and Data Security

The survey was prefaced with an introduction sheet explaining the purpose of the survey, the researchers involved, and addressed ethical issues associated with informed consent, privacy and data security (see Supplementary Material). All work associated with this survey was conducted under Griffith University Ethics reference number 2020/982.

## Results

### Expert Demographics

Forty-six experts responded to the survey. Most respondents were based in Queensland (37%) or Victoria (22%), with fewer respondents from other states (4–11%) and no respondents from Tasmania (Table S1). Most worked for government agencies (48%) or academic institutions (24%) as researchers (39%) or conservation/land managers (33%). Almost one-quarter worked in policy development/implementation or fire management. Respondents generally had many years of experience, with most (72%) being in their current roles for at least 10 years, and almost 40% for over 20 years. Roughly three-quarters of respondents reported having direct experience with both fire management and the impacts of natural fires in ecosystems.

### Expert Knowledge of and Experience with Wildfire Management

To determine respondent’s level of expertise in wildfire management and their subsequent ability to identify key knowledge gaps and research priorities in relation to FFC use, we asked respondents about their knowledge of and experience with eight standard fire management practices, including (1) aerial fire-fighting chemical (FFC) deployment, i.e., physical deployment of chemicals from aircrafts, (2) aerial fire-fighting flight planning, i.e., planning flight paths, (3) applied research into fire management, (4) controlled burn planning including timing, location, staffing, equipment, logistics, (5) on-ground controlled burns management, (6) fire-fighting planning for unplanned burns (natural fires) including timing, location staffing, equipment, logistics, (7) on ground fire-fighting of unplanned fires, i.e. back-burning, FFC deployment, and (8) selection of FFCs used in fighting/managing fires.

Roughly 60% of respondents had experience with controlled burns planning and management, with approximately 25% stating they had extensive experience with both (Fig. S1). Similarly, approximately 60% of respondents had experience with unplanned fires, in relation to both planning and logistics and on-ground fire-fighting. Over 30% reported medium to extensive experience in these scenarios. Few respondents ( < 30%), however, had experience with aerial fire-fighting, either with chemical deployment or flight planning. Despite their involvement in on-ground fire-fighting and management and relatively high levels of experience in applied fire management research (65% reported at least some experience), 80% of respondents reported having little or no experience selecting the FFCs used.

### Expert Knowledge of and Experience with Impacts of Wildfires

Respondents were asked about their direct experience with six common fields associated with the impacts of fire, including (1) applied research into the impacts of fire on biodiversity, (2) emergency species translocations—salvage translocations, (3) post-fire clean-up, (4) post-fire monitoring of abiotic environmental quality, e.g. erosion, soil nutrient levels, (5) post-fire monitoring of species and habitat recovery, and (6) post-fire monitoring of water quality including sedimentation and nutrient levels.

Almost 70% of respondents reported being involved in applied research into the impacts of fire on biodiversity (Fig. S1), including co-ordination of ecological post-fire recovery projects. Most of these respondents were academics and/or land managers. Conversely, few respondents ( ~ 20%) had any experience with emergency species translocations (relocations of animals) after fires, with only two respondents having extensive experience in this area. Roughly 60% of respondents had post-fire clean-up experience, most of whom worked for local or state government and fire services. Most respondents had experience with post-fire environmental quality monitoring (68%) or species and habitat recovery (75%). Fewer respondents (56%) were involved in monitoring water quality after fires.

### Environmental Concerns about Wildfires

To obtain an overview of the primary environmental concerns relating to wildfires in Australia, experts were asked to indicate their levels of concern from 1 (not concerned) to 5 (very concerned) about six fire-related environmental issues. These included (1) the efficacy of fire management policies, e.g. local government fire plans, National Fire Service or Rural Fire Service policy, (2) the use of current fire management strategies, including controlled burns, and their impacts on species and ecosystems, (3) impacts of fire on species and ecosystems, (4) impacts of fire on the abiotic environment, e.g. erosion, nutrient cycles, water quality, leachates, (5) the use of FFCs and their impact on species and ecosystems, and (6) the combined effects of fire and other environmental stressors including disease, drought, pollution and climate change, on species and ecosystems.

**Fig. 1:**
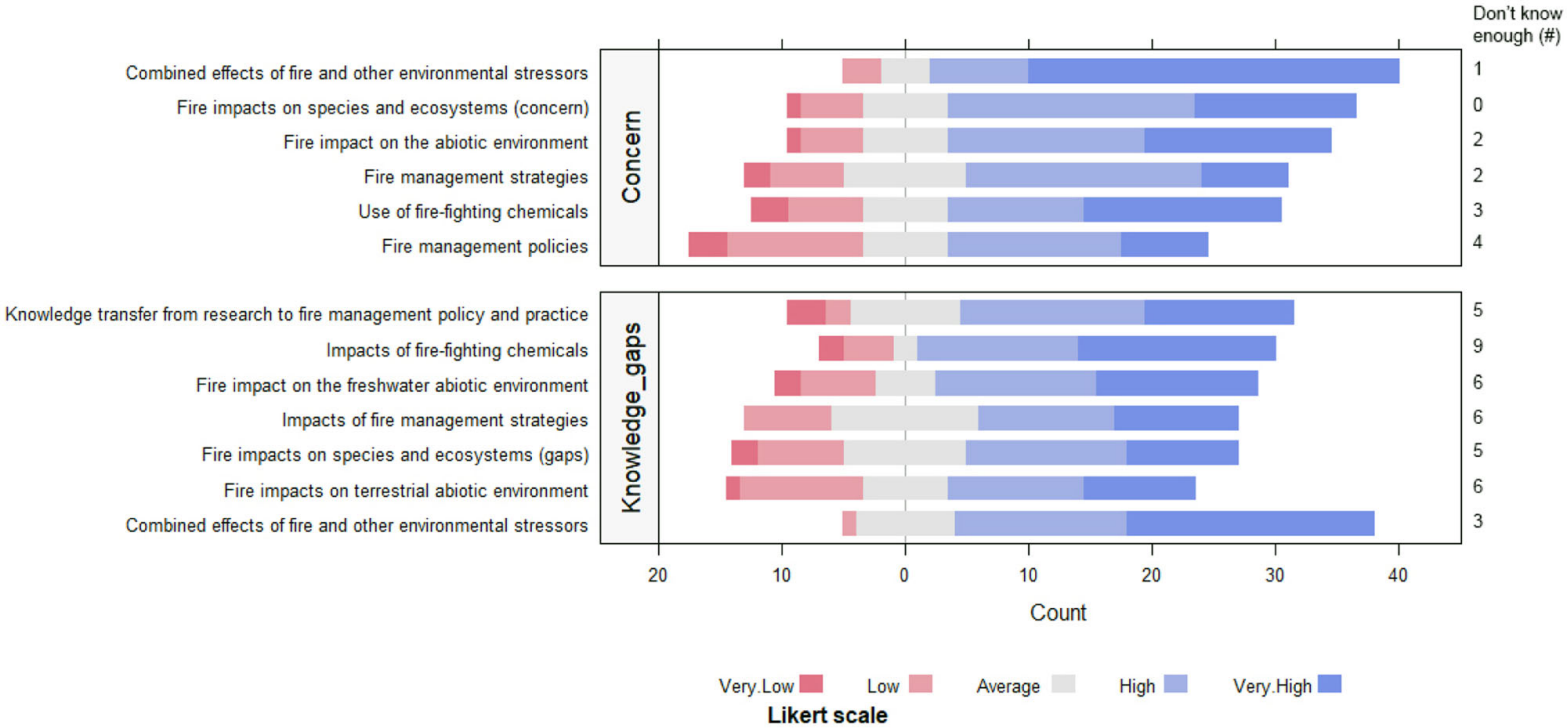
Levels of environmental concern about fire-related issues in Australia (top) and Respondent rating of key knowledge gaps about fire management and environmental impacts in Australia (bottom)

Respondents were concerned about all six issues (Fig. 1a), particularly the combined effects of fire and other environmental stressors (mean score 4.35 ± 1.11, 65% very concerned), the impacts of fires on species and ecosystems (3.84 ± 1.13, 28% very concerned) and the effects of FFCs on biodiversity (3.48 ± 1.54, 35% very concerned). In some cases, respondents indicated that they did not know enough about an issue to accurately rate their level of concern.

Several respondents mentioned that many areas have a long history of fire events and management and that fires are not always destructive. One respondent further elaborated on the importance of the fire as an ecological process in Australia, *“I think it’s important to make a distinction between fires (bushfires or planned fires) that occur within the limits of an ecosystems ‘normal’ fire regime and those that occur beyond this. Planned fires can achieve amazing ecological outcomes if they are in keeping with the natural fire regime. Important not to demonize fire - it’s a very important ecological process.”* (Land Manager, South Australia).

### Knowledge Gaps in Relation to Wildfire Management and Environmental Impacts

To identify key knowledge gaps concerning wildfire management and environmental impacts in Australia, we asked respondents to rate how concerned they were about knowledge gaps in seven areas, (1) impacts of fire on species and ecosystems in general, (2) impacts of fire on the freshwater abiotic environment, e.g. water quality, leachates, sedimentation, (3) impacts of fire on the terrestrial abiotic environment, e.g. soil erosion, soil nutrient cycles, leachates, (4) knowledge transfer from research to fire management policy and practices, including local government fire plans, National Fire Service or Rural Fire Service policy, (5) impacts on species and ecosystems of fire management strategies such as frequency, timing and location of controlled burns, (6) impacts of FFCs (aerial and on ground deployed) on species and ecosystems, and (7) combined effects of fires and other stressors, e.g., disease, drought, climate change, on species and ecosystems.

Respondents were concerned about knowledge gaps in all seven areas (Fig. 1b) but were particularly concerned about the combined effects of fire and other stressors (mean score 3.95 ± 1.32, 74% concerned to very concerned), impacts of fire on the freshwater abiotic environment (3.34 ± 1.59, 56% concerned to very concerned), knowledge transfer from research to fire management policy (3.22 ± 1.71, 59% concerned to very concerned), and impacts of FFCs (3.22 ± 1.91, 63% concerned to very concerned). In several cases, respondents indicated that they did not know enough about the issue to make a judgment, further highlighting knowledge gaps among experts surveyed.

In addition to these concerns, several respondents highlighted knowledge gaps associated with natural fire regimes, the impacts of a lack of fire on ecosystem diversity, and how climate change will affect fire regimes, and therefore affect species and ecosystems.

*“Issues relating to the lack of fire in some plant communities and the loss of biodiversity. The challenge of managing populations in fragmented landscapes where fire is being used to manage habitats. The impacts of total grazing pressure on post fire habitat recovery.”* (Conservation Manager, South Australia)

*“Fire ecology is a very new discipline of science. We’ve learned a lot but there are still very significant knowledge gaps in every area. I think the most significant gaps are in understanding how species respond to changes in fire regime and how we can manage this with a changing climate.”* (Land Manager, South Australia)

### Knowledge of Fire-fighting Chemicals

To collate information on expert knowledge of FFC use in Australia, we asked respondents about (1) their familiarity with 12 common FFC brands and formulations, (2) what ecotoxicological and environmental impacts of FFCs they were familiar with, such as acute and chronic lethal toxicity, bioaccumulation, or biochemical oxygen demand changes, (3) what impacts of FFCs they were concerned with, (4) which fauna groups they were concerned about in relation to FFC use, and (5) which interactions with FFCs they were concerned about for species and ecosystems, e.g., pollution, bushfire leachates/sediment, climate change, or disease.

**Fig. 2:**
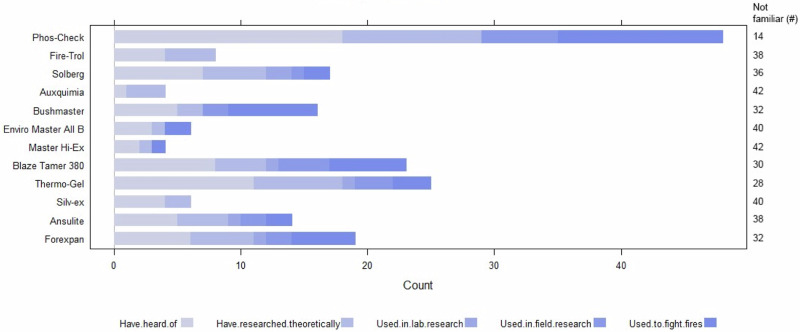
Respondent’s familiarity with different fire-fighting chemical brands and formulations (Note: categories were not exclusive)

Most respondents were unfamiliar with many of the FFCs in the survey (Fig. 2). Phos-Check (70% familiarity), Thermo-Gel (39%), Bushmaster (31%) and Blaze Tamer (35%) were the most well-known brands/formulations, but knowledge was often limited to theoretical knowledge gained through the literature, media, or conferences, with minimal practical experience (used to fight fires, field-based research, lab-based research). Unsurprisingly, respondents working in land management or fire management roles had the greatest practical familiarity with a broader range of FFCs (8 of the 12 FFCs). Two fire management participants also identified several other FFCs that they were familiar with, including *Aquagel K, Insul8, WD-881, FireIce* and *Baricade* (Fire Manager, Northern Territory; Fire Manager, Australian Capital Territory).

**Fig. 3:**
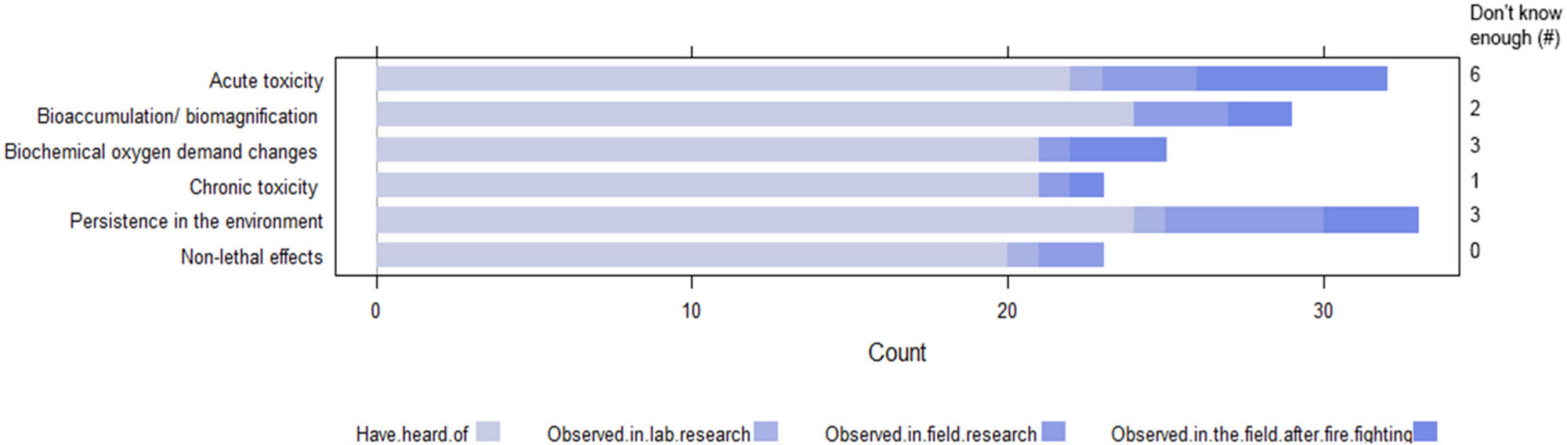
Respondent’s familiarity with the impacts of bushfire-fighting chemicals (FFCs)

Some participants indicated that they had direct familiarity with the effects of FFCs in the field (either after fighting fires or field-based research) or in laboratory settings (Fig. 3), which consisted primarily of acute toxicity, bioaccumulation and environmental persistence. Most participants indicated that they were indirectly familiar with the effects of FFCs due to reading the literature, attending conferences, and through the media, but a large number stated that they did not know enough about the impacts (32–54% unfamiliarity).

**Fig. 4:**
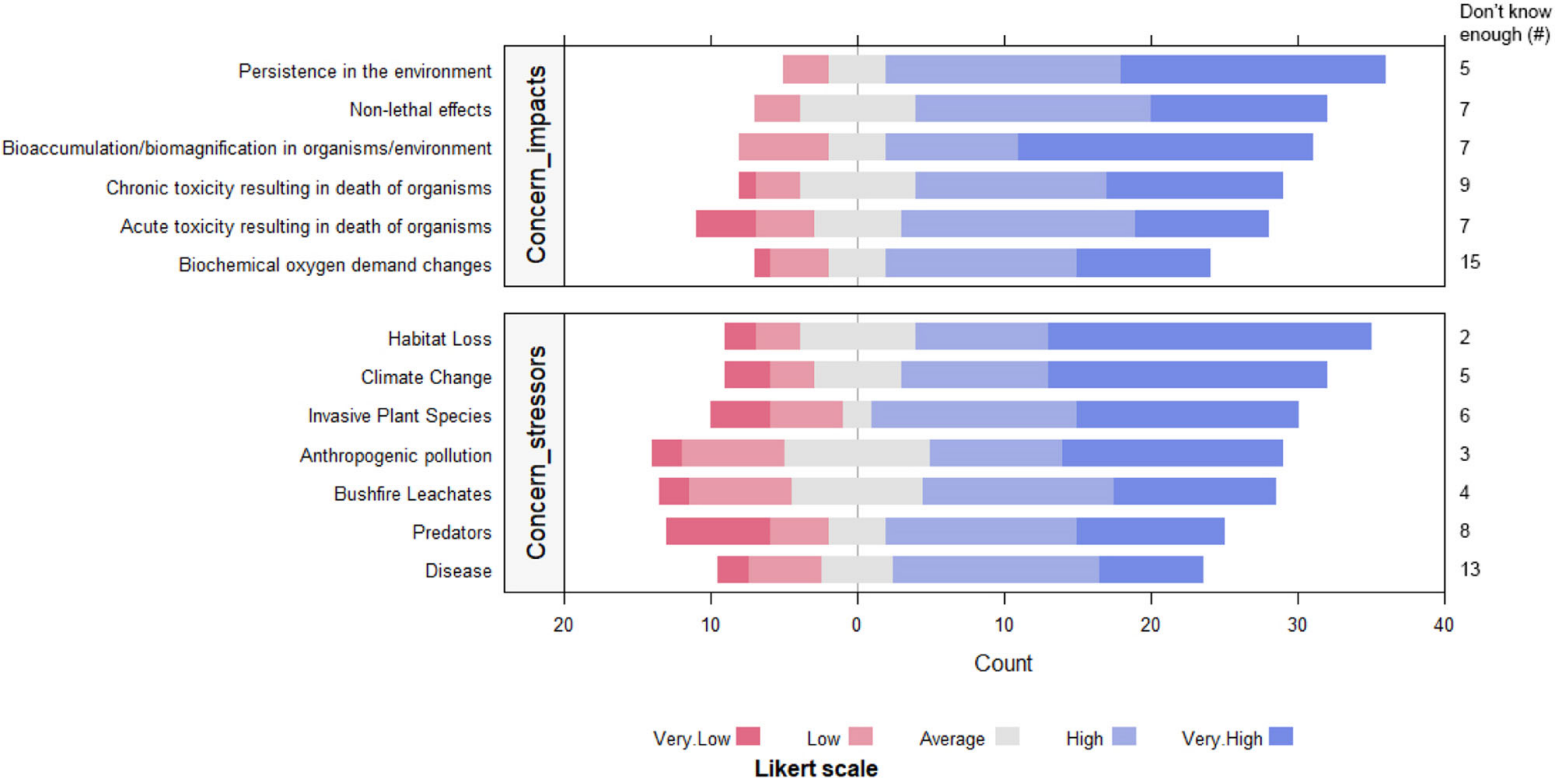
Respondent’s concerns about the potential impacts of FFCs on Australian species and ecosystems (top), and potential stressor that may exacerbate the impacts of FFCs on Australian species and ecosystems (bottom)

Most respondents were highly concerned about the impacts of FFCs (average > 3), with the highest levels associated with FFC persistence in the environment (3.74 ± 1.55, 39% very concerned), bioaccumulation/biomagnification (3.48 ± 1.79, 44% very concerned), and non-lethal effects (3.35 ± 1.64, 26% very concerned) (Fig. 4a). The highest levels of uncertainty were associated with changes in biochemical oxygen demand (33% did not know enough to make a judgment) and chronic toxicity (20%). One participant provided an example of what they believed to be an impact of FFCs on an aquatic system, *“As a member of [redacted], we dropped considerable amounts of Phoschek in the Warragamba catchment basin, which subsequently suffered a significant algal bloom. Not normally experienced in that catchment.”* (Fire Manager, Queensland).

**Fig. 5:**
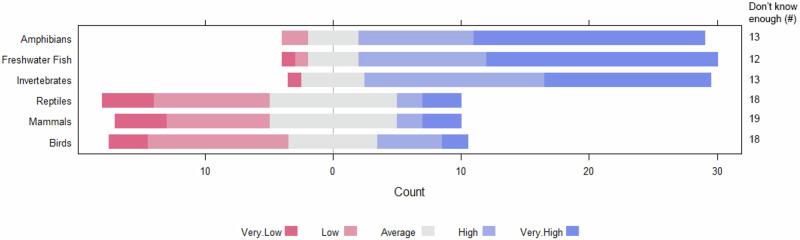
Respondent’s assessment of the potential impacts of FFCs on different taxonomic groups

Respondents were most concerned about the effects of FFCs on freshwater fish (3.15 ± 2.05, 39% very concerned), amphibians (3.09 ± 2.08, 39% very concerned) and invertebrates (2.98 ± 2.02, 28% very concerned) (Fig. 5). While less concern was associated with impacts on mammals, reptiles and birds, this may be due to the high levels of uncertainty (‘do not know enough to make a judgment’) for these groups at 41%, 39% and 39%, respectively.

When asked to identify individual fauna species or taxonomic groups of concern within aquatic systems, and why they were of concern, 21 respondents provided more detail. Ten respondents identified amphibians as being vulnerable due to toxin absorption through the skin (frogs) or bioaccumulation of compounds in the system (tadpoles). Macroinvertebrates, including freshwater crayfish, freshwater mussels and other benthic species, were identified by eight respondents due to the effects of acute toxicity, bioaccumulation, lower biochemical oxygen demand, and the non-migratory nature of many of these species. Freshwater fish, particularly non-migratory species and those occurring in upland, isolated waterways such as Galaxiids, were also identified by 7 respondents. Some respondents highlighted other species, such as platypus, turtles and birds, due to the bioaccumulation of chemicals in the tissues of lower-order prey species. In some cases, respondents indicated concern for species found in specific aquatic habitats, such as upland streams and swamps, lentic systems with no water flow, and shallow streams. One respondent also indicated concern about the impacts of FFCs on flora, “*Many Australian plants cannot tolerate exposure to phosphorus (some cannot tolerate any exposure) -- I believe DAP was a key fire retardant used in the bushfires and the impact of these on Proteaceae is not clear to me*” (Academic, Australian Capital Territory).

When asked to identify which additional stressors were likely to exacerbate the impacts of FFCs on species and ecosystems, participants indicated that they were most concerned about habitat loss (3.87 ± 1.41, 47% very concerned), climate change (3.52 ± 1.70, 41% very concerned) and pollution (3.41 ± 1.49, 33% very concerned) (Fig. 4b).

Several participants provided additional details or examples of how they anticipated other stressors would exacerbate the impacts of fire-fighting chemicals or vice versa, *“My concerns with regards to retardants relate largely to the effects that these chemicals are likely to have on habitat condition (due to changes in plant growth - particularly weed invasion and waterway nutrification) and the additive impacts this will have when combined with habitat loss, etc. With regards to foams used in fire-fighting, I would be more concerned about the impacts these have on frogs, aquatic organisms and some invertebrates (those without an exoskeleton) because they will make them more prone to disease and other impacts.”* (Land Manager, South Australia).

*“The addition of nutrients to natural areas in the form of fire-fighting chemicals is of concern as there are plenty of papers on how the addition of nutrients can degrade natural areas and the effects can be long lasting. For example, it may promote weed growth that degrades habitat into the long term. There are numerous threatened plant sites where the importance of not adding nutrients, including in the form of fire retardants and foams, is stressed when undertaking activities such as blackening out the edges of burns, etc.”* (Land Manager, South Australia).

One participant suggested that current fire-fighting practices and the chemicals used did not support concerns about the impacts of FFCs on natural systems, *“The composition of firefighting agents for bushfires (not including PFAS Ansulite) are fully biodegradable and used at low concentrations dispersed along limited tracks of the fire front with riparian and water courses avoided. They are not intensively applied over square kilometers. No significant effects are expected, no significant effects have been seen on water.”* (Policy Developer, Queensland).

### Organizational Needs for Management of Fire-fighting Chemicals

Finally, we asked the respondents in an open-ended question to identify the information that they or their organization needed to help manage their activities in relation to FFC use and their potential ecological impacts. Twenty-five respondents provided detailed responses to this question which we broadly grouped into four main categories based on common themes in the responses: ecotoxicology information, cost of FFC use, knowledge dissemination, and FFC use recommendations (Table 1).

**Table 1:** Organizational informational needs for management of fire-fighting chemicals (FFCs) identified by expert respondents

Theme	Description/justification	Examples of specific information needs identified by experts
Ecotoxicology: Acute or chronic effects of FFCs on aquatic fauna and systems	More detailed and specific information on half-life, decomposition, bioaccumulation and toxicity rates for all currently used FFCs in Australia	• What are the LC50 and LD50 concentrations of FFCs? • Is toxicity ameliorated by exposure on the ground? • Does toxicity vary instream with sediments or tannins? • How far downstream from sites of application does toxicity last? • Do the different types (brands) and forms (e.g., gels vs. foams vs. retardants vs. drip torch) of FFCs have different impacts on species and ecosystems? • What are the potential risks of FFC bioaccumulation? • What are the potential risks to higher-level trophic organisms through biomagnification? • What are the relative impacts of FFCs on water quality in drinking water catchments and do these impacts only occur at certain doses or with certain FFC types?
Cost of FFC use	Cost-benefit analyses comparing the use of FFCs vs. pre-emptive actions to keep fires small will support decision-making and policy in relation to fire management at the landscape scale and over longer timeframes	• What is the cost of using FFCs vs. pre-emptive management actions, including control burns or intense helicopter water bombing?
Knowledge dissemination	Better dissemination of and access to accurate and up-to-date scientific information on FFCs for decision-making, including dissemination channels and open access publications	• What are the best channels for dissemination of information on the impacts of FFCs for different stakeholders? • How do we best translate scientific research into management actions or recommendations?
FFC recommendations	Suitability of different fire-fighting chemicals for application in different habitats or environments	• Can we develop a rating system for the use of different FFCs in different habitat types, e.g., organic soils, sand-based environments, peatlands, and including in protected areas where there are fewer other sources of potential contaminants (particularly upland protected areas)?

Two respondents indicated that there was no additional information that they required and that what they needed to know was already publicly available, *“I am satisfied with the level of information. I suspect the problem is cultural and the way fire is managed more broadly.”* (Fire Manager, South Australia). *“There is good information in Safety Data Sheets, and I have confidence in the USFS QPL process.”* (Academic, Australian Capital Territory).

## Discussion

While large and intense wildfires are an integral part of many landscapes and can play a critical role in ecosystem dynamics (Bargali et al. 2023; Bargali et al. 2024; Bond and Keeley 2005; Erni et al. 2024; Verma and Jayakumar 2012), increased fire season length, reduced duration between fire intervals, and greater fire intensity have significant ecological impacts as well as far-reaching economic and social implications. Fire management in Australia and globally is a complex issue involving multiple stakeholders with different priorities, knowledge and resources across varied landscapes and geographic areas subjected to different regulatory policies (COAG 2004; Law et al. 2022; McDonald and McCormack 2022; Stephens 2011). The 46 experts surveyed in this study reported knowledge of and experience with the impacts and management of wildfires in Australia. Unsurprisingly, some groups self-identified as having more knowledge and experience in some areas than others. For example, land managers and those working in fire management roles claimed more experience and knowledge of the different FFCs used in Australia to manage planned (i.e., control) and natural (i.e., wildland) fires and their impacts in the field. Those in academic roles claimed more experience determining the environmental and biological effects of fire in lab- and field-based settings, while those working in government policy roles were better able to comment on the efficacy of current fire management policy in relation to FFC use.

Despite different areas of expertise, there was consensus about the most pressing concerns in relation to fire management and the ecological impacts of bushfires. These included the effects of fire on fauna and ecosystems (such as the extent of habitat loss and direct and indirect fauna loss) and the combined impacts of fires and other stressors such as pollution, climate change and disease. Many of these concerns have been highlighted in the literature (Beranek et al. 2023; Dickman 2021; Hayward et al. 2022; Kemter et al. 2021; Nolan et al. 2021), have been the focus of several funding programs (e.g., the Federal government A$200 M Wildlife and Habitat Bushfire Recovery program, (DECCEEW 2023)), are included in Australian government inquiry documents e.g., SFPARC’s “Lessons to be learned in relation to the Australian bushfire season 2019–2020”; (SFPARC 2020; Khan 2021), and/or included in environmental management policy.

FFC use was in the top three concerns about the impacts of fire on native fauna and ecosystems, with majority of experts indicating that FFC impacts were of increasing concern in the face of future wildfire frequency and intensity predictions. While all states have moved to ban the use of Per- and Poly-fluoroalkyl (PFAS) compounds in FFCs in Australia, concern about the effects of newer “safer” FFCs is reflected in some Australian policies. For example, the Queensland Firefighting Foam Management Policy states that “*All foams have potential adverse effects, with risks that are specific to the foam type, situation and location. Depending on the foam type and composition, the chemicals used can have short and long-term impacts on biota, soils and waterways and other values through their persistence, bioaccumulation, toxicity and oxygen demand*.” (DES 2021). The Victorian EPA factsheet on fire retardants and health highlights that fire retardants, and especially foams, have been shown to have impacts on native plants and aquatic species and, therefore, pilots are advised not to apply retardants close to waterways (Victoria EPA 2020).

While two respondents indicated that they were satisfied with the information currently available for FFCs through the FFC Safety Data Sheets (SDS) produced by the United States Forestry Service Qualified Products List Process (USDA n.d.), the other 44 respondents stated that several key knowledge gaps needed to be addressed to enable them and their organizations to better manage FFC use in the future. The Queensland Department of Environment, Tourism, Science and Innovation (DETSI) recognises the potential inadequacy of relying on SDS to assess FFCs in its *Procedural Guide on the Use of Bushfire Firefighting Agents* for National Parks estate and for vegetation fires across Queensland more broadly, stating that “*In assessing particular products, it is worth noting that safety data sheets (SDS) for many products can be unreliable, inadequate and even misleading and so should not be relied upon as the main source of product information and effects. The SDS for many chemicals including firefighting products can be particularly deficient in information relevant to environmental issues*.”

### Key knowledge gaps and research priorities in relation to FFC use and environmental impacts in Australia

The experts identified several key knowledge gaps on the use and effects of FFCs on fauna and ecosystems. These included a paucity of knowledge on:

1. The different types of FFC formulations currently used in Australia, and the potential use of PFAS-containing chemicals in some states in response to ‘catastrophic events’ (e.g., NSW EPA, 2020)
2. The short and long-term ecological impacts of current FFC formulations, including direct and indirect effects on all levels of food webs and ecological communities
3. The exacerbative interactions between FFCs and other environmental stressors
4. The short and long-term impacts of FFCs on abiotic water system characteristics, including in drinking water catchments

The poor dissemination of current knowledge from researchers to other stakeholders, including policy planners, local and state fire services, local government and other land managers, was also highlighted as an important gap. Solving complex socio-environmental challenges, such as wildfire management, requires the uptake and integration of scientific knowledge and evidence into decision-making processes (Sutherland et al. 2004). This translation of knowledge into action sees research findings taken up by end-users and used appropriately to inform policy, practice or further research (Lundmark et al. 2023; Tuohy et al. 2024). Knowledge transfer across the science-policy gap and between stakeholders dealing with large transdisciplinary issues is commonly cited as a key obstacle to the development and implementation of science-based best-practice policy to support on-ground action (Fleischman and Briske 2016; Hughes et al. 2016; Nyssa 2020; Wistbacka et al. 2018). Some respondents highlighted prioritizing the publication of key information in Open Access journals to enable local government and other practitioners and policy makers to access important information in a timely manner (sensu Roche et al. 2021), while others suggested including environmental impacts of FFCs in Rural Firefighter Association training manuals and sessions.

Given the key knowledge gaps highlighted by the experts (including end-users of the knowledge) in our study, three initial research priority themes can guide program direction and funding support for FFC use in Australia. These themes are centered around quantitatively assessing the ecological impacts of FFCs, primarily in aquatic systems. More specifically, the experts articulated that research should be focused on the following:

1. ***Determining the environmental persistence and toxicity of all currently used FFCs in an Australian context***, including the effects of different types of the FFCs used (i.e., foams, retardants, gels) on native freshwater fauna species, including frogs, fish and macroinvertebrates.
2. ***Assessing longer-term multigenerational impacts of FFCs****,* including threats to higher trophic order organisms through biomagnification.
3. ***Evaluating the combined and interactive effects between FFCs and other environmental stressors****,* including habitat loss, anthropogenic pollution, bushfire leachates and climate change.

The few studies published since the reviews by Adams and Simmons (1999) and Gould et al. (2000) on the impacts of commonly-used FFCs on Australian vegetation, stream macroinvertebrates and tadpoles in field and laboratory settings contribute to addressing these research priorities (e.g., Bell et al. 2005; Boulton et al. 2003; Tunstill et al. 2022). These studies also highlight the importance and need for further targeted research to capture the extent and significant complexity of real-world FFCs deployment in future environmental impact assessments. Our study highlights the need for concerted research (and associated funding) for transdisciplinary programs that meet national and global environmental projection goals. Collaboration between stakeholders, including academic researchers, conservation/land managers, fire and disaster management agencies, and policy makers, amongst others, is needed to ensure that the knowledge generated meets the needs of end-users, is transferred between all stakeholders, and provides science-based evidence to inform FFC policy and strategy development. Involving end-users and practitioners will ensure that implementing the subsequent policy recommendations is feasible in practice and considers the different stakeholders, federal, state or local government contexts and the complexity of the interactions between them (Clare and Creed 2022).

### Limitations of study

It is important to note that the professional roles, expertise and geographical location of survey respondents varied and that the numbers of respondents were not always equivalent between categories (Table S1, Fig. S1). As such, not all views were captured in this study, and other priorities and knowledge gaps relating to FFC use may remain unidentified, including the impacts of FFCs on flora and vegetation communities which were not explicitly examined in this study. However, given the strong consensus about key knowledge gaps and research priorities from most experts surveyed, this study provides a critical first step in identifying FFC research priorities for practitioners and policy makers that can be addressed in parallel to research examining the extent and the social, economic and environmental impacts of wildfires in Australia. While this study is restricted to the Australian context, the methods used and information collected will be useful to stakeholder groups working with FFCs to manage wildfires in other countries more broadly facilitating the identification of global knowledge gaps and research priorities.

## Supplementary information

Supplementary Material

## Data Availability

No datasets were generated or analysed during the current study.
