# Supplementary material for: Impacts of Fire-fighting Chemicals on Native Fauna and Ecosystems in Australia: Identification of Key Knowledge Gaps and Research Priorities
Source: Environ Manage. 2025 Mar 24;75(6):1559–70. doi: 10.1007/s00267-025-02143-z (PMC12084226; doi:10.1007/s00267-025-02143-z)
Supplement: Supplementary file 1 — Supplementary Material [file 267_2025_2143_MOESM1_ESM.docx]

# Supplementary Material

## Online survey and participant information sheet

**Impact of fire-fighting chemicals on species and ecosystems**

Dear Participant,

Climate change is leading to drier and warmer weather conditions which in turn lead to longer and more intense fire seasons and more frequent and/or severe fires in Australia. These fires have significant impacts on biodiversity and a range of strategies and policies are in place to manage the impacts of fire on species and ecosystems. One of the main strategies in place to fight natural fires is the use of fire-fighting chemicals. Increasing fire frequency and severity associated with predicted future climate change will lead to increased use of these chemicals in the natural environment. To date, however, little is known about the impacts of fire-fighting chemicals on Australian species and ecosystems.

The purpose of this research is to collect expert opinion on the impacts of fire-fighting chemicals on species and ecosystems and identify key knowledge gaps and research priorities in relation to fire-fighting chemical use in Australia.

This work is being conducted by Dr Clare Morrison and Dr Laura Grogan from the School of Environment and Science and Dr Chantal Lanctôt from the Australian Rivers Institute, Griffith University as part of a larger study into the impacts of fire-fighting chemicals on Australian fauna.

The anonymous online survey includes close-ended (Likert-scale) and open-ended questions and it is anticipated that the survey will take approximately 15-20 minutes to complete. Participation in this study is completely voluntary. However, you will not be able to withdraw from the study once you have submitted the answers to the questionnaire. Given the anonymous nature of the data, it will not be possible to identify individual responses to remove them.

The information obtained from the survey will be dealt with in a secure manner and your responses will remain anonymous. All research data (responses and analysis) will be retained in a password protected electronic file at Griffith University for five years before being destroyed.

Griffith University conducts research in accordance with the National Statement on Ethical Conduct in Human Research. If you have any concerns or complaints about the ethical conduct of the research project please contact the Manager, Research Ethics on 3735 4375 or [research-ethics@griffith.edu.au](mailto:research-ethics@griffith.edu.au). The Griffith University Ethics reference number for this project is GU ref no: 2020/982.

For more information, please contact Dr Clare Morrison ([c.morrison@griffith.edu.au](mailto:c.morrison@griffith.edu.au))


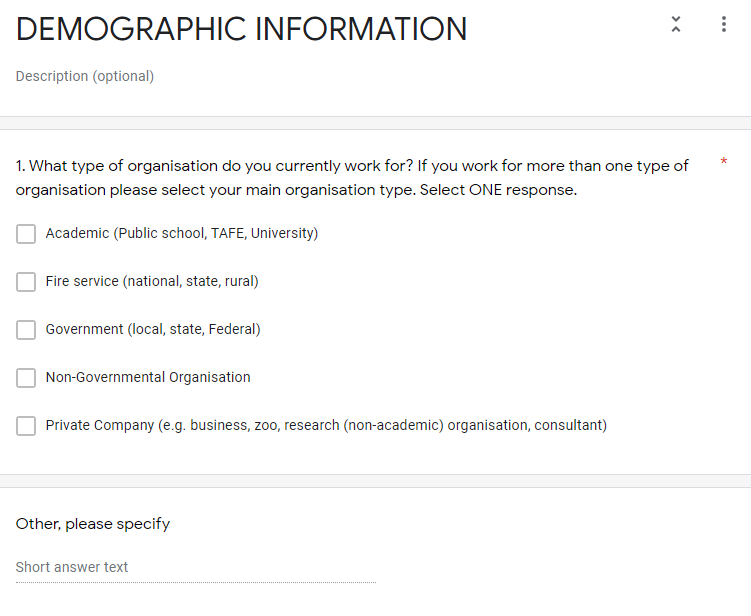

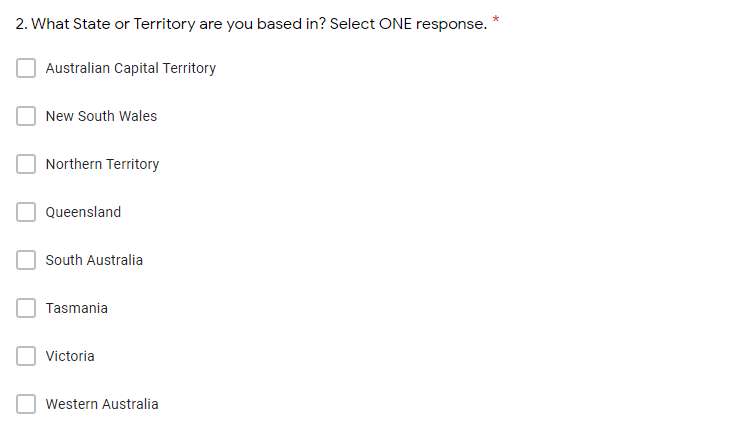


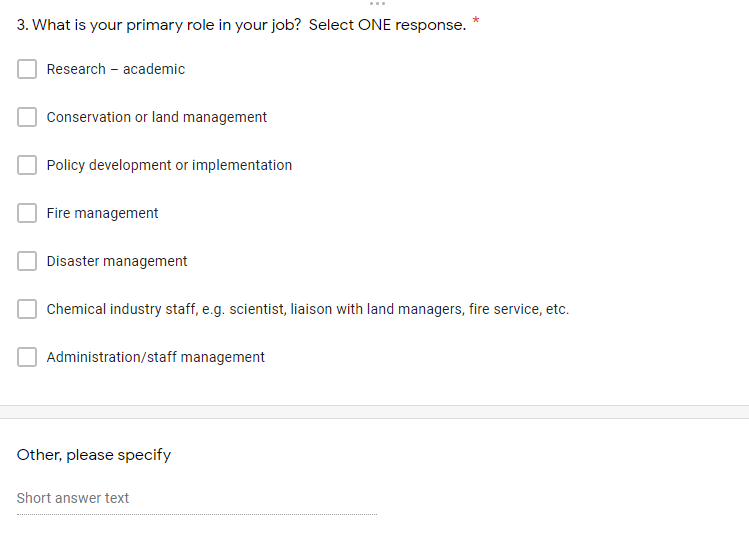

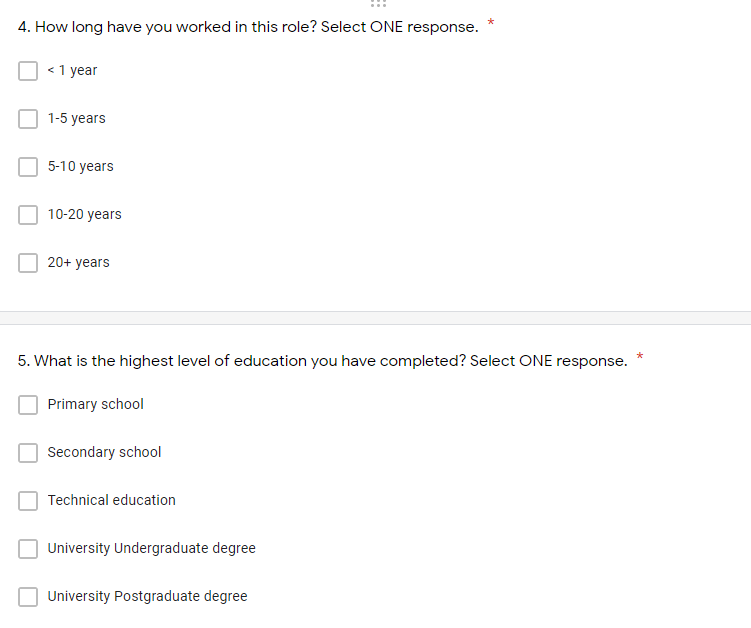


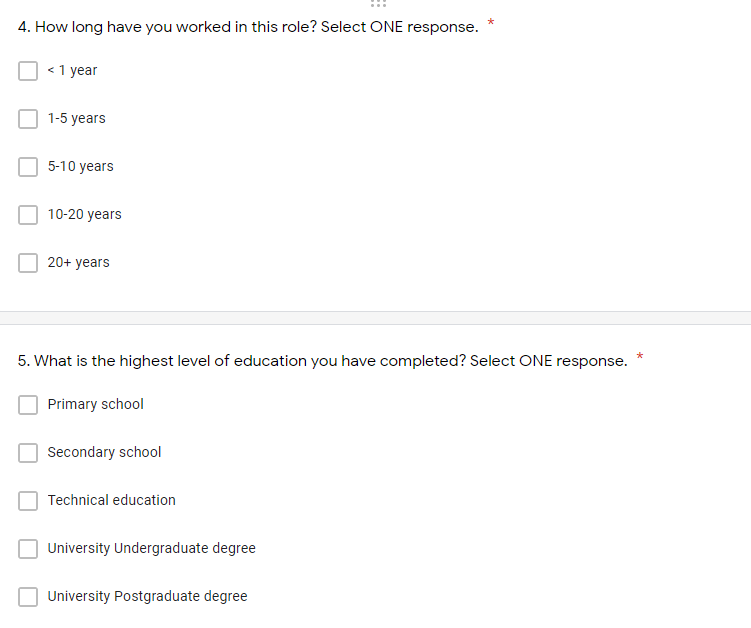

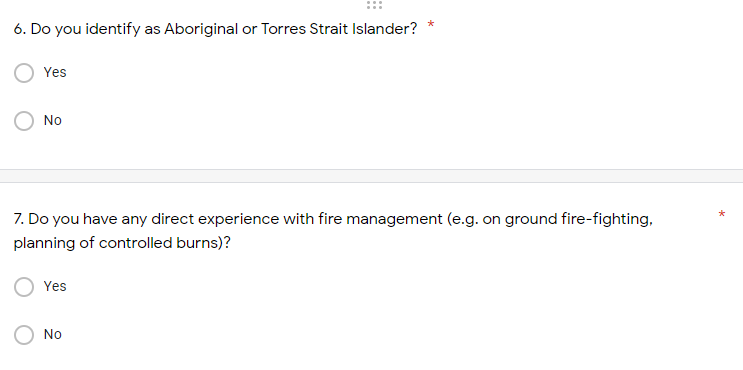

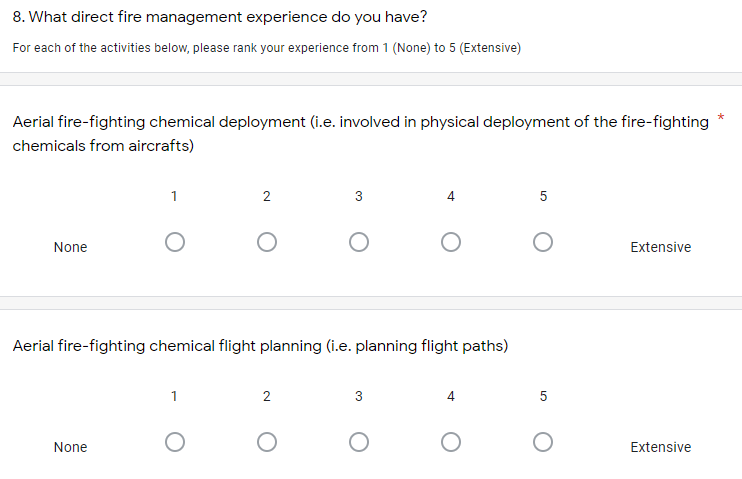


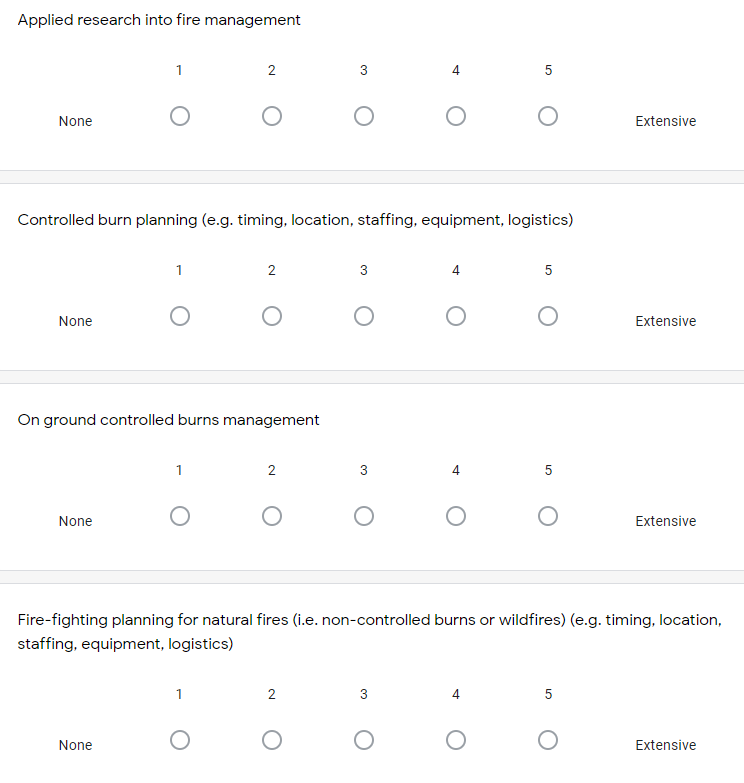

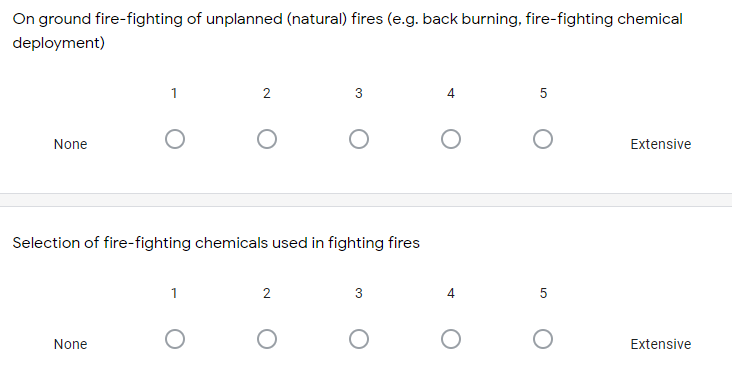


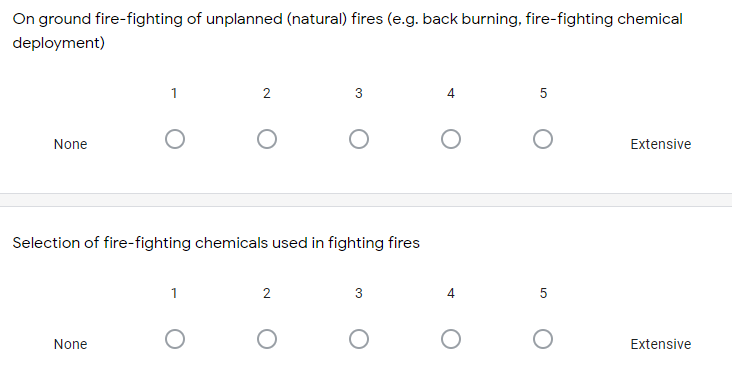

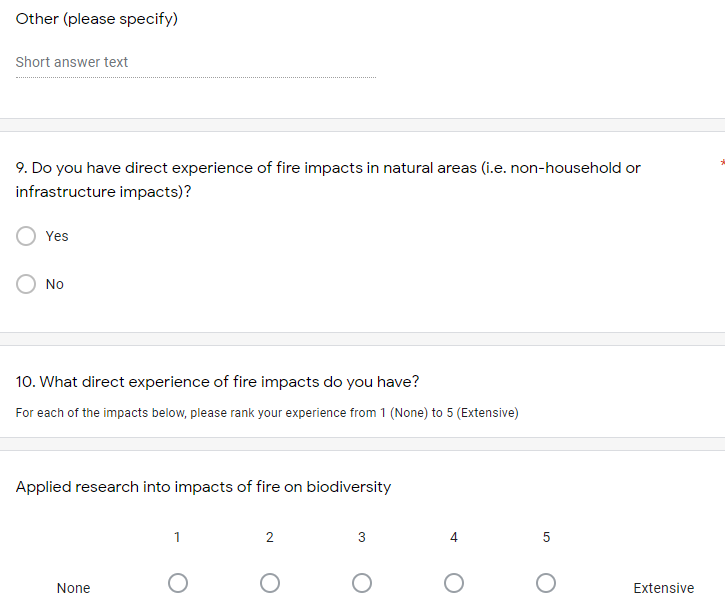

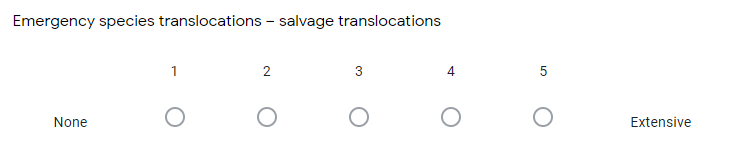

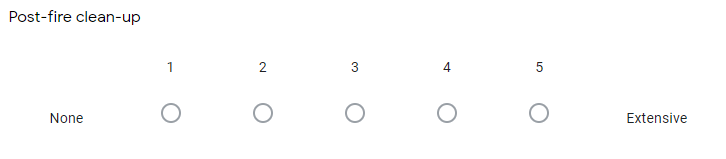


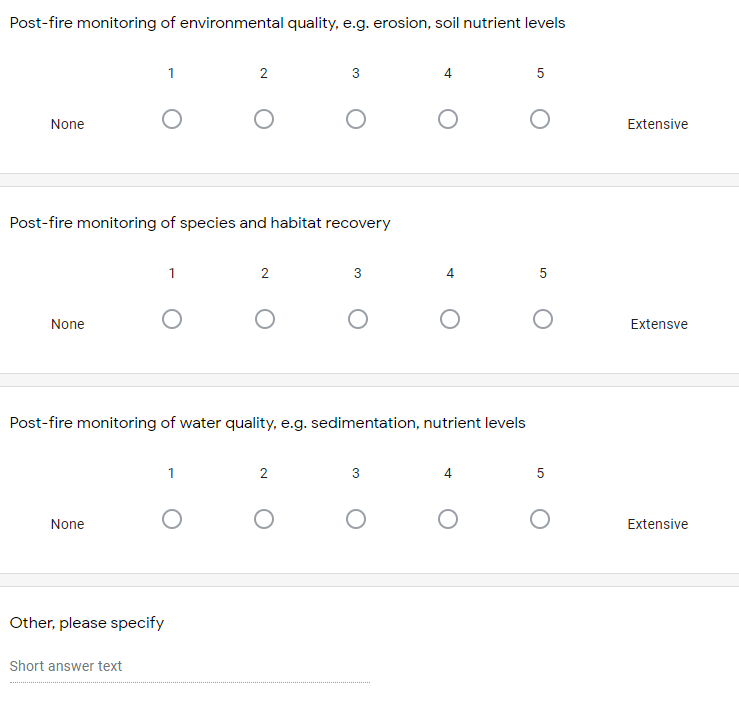


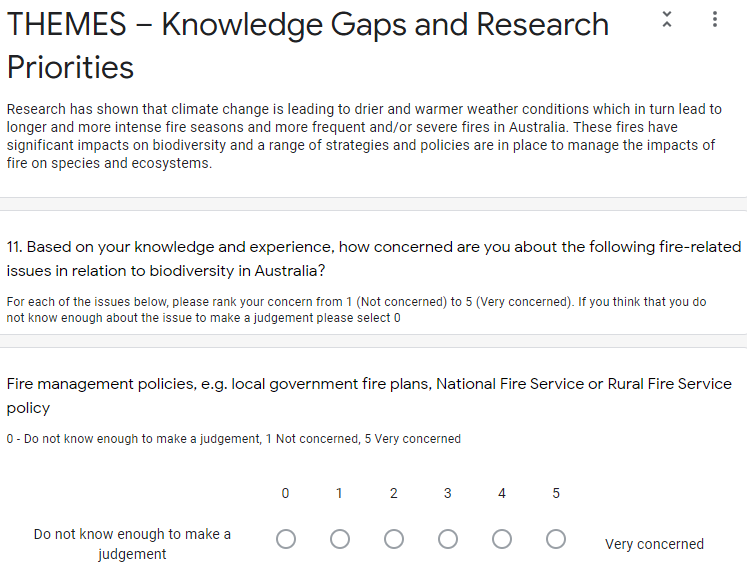

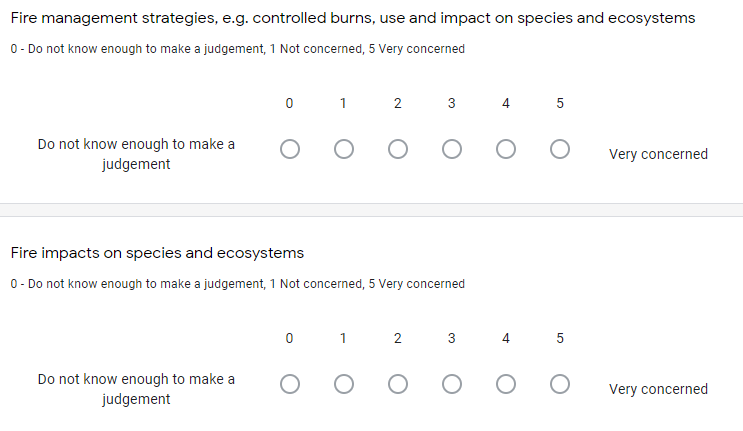


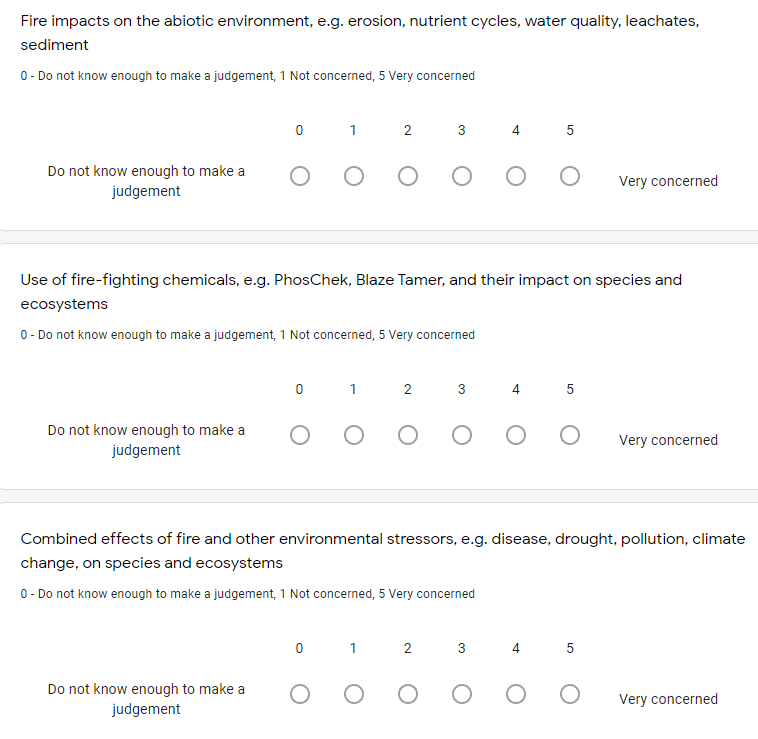

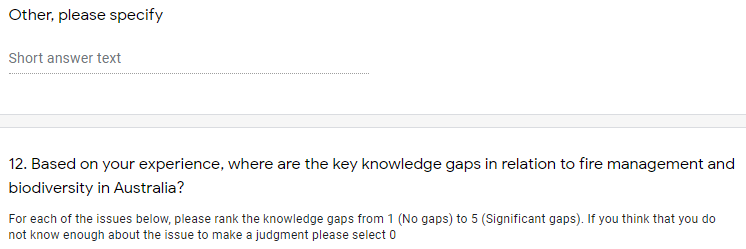


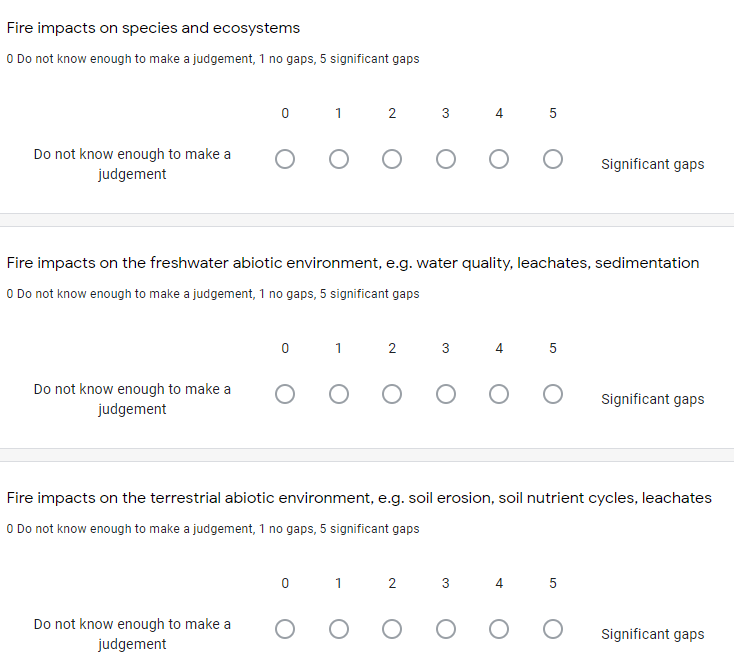


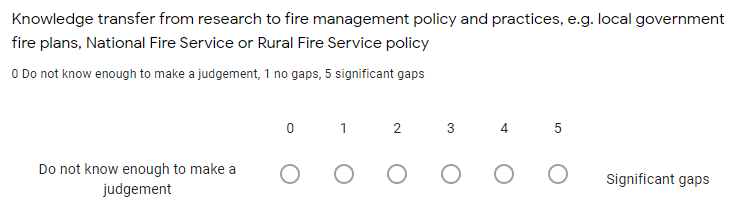

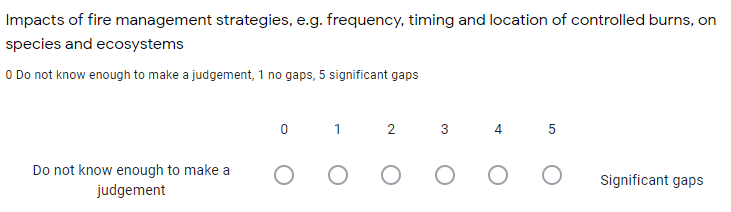


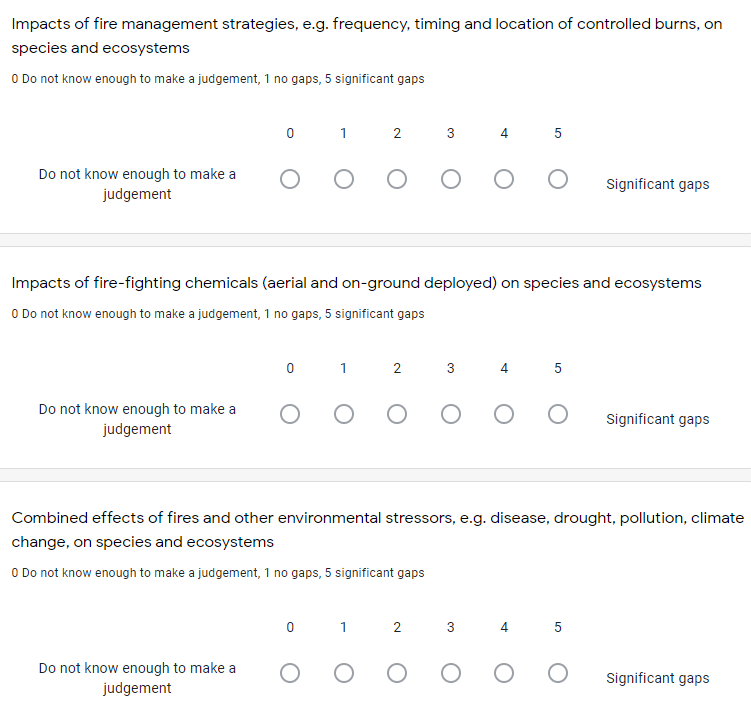


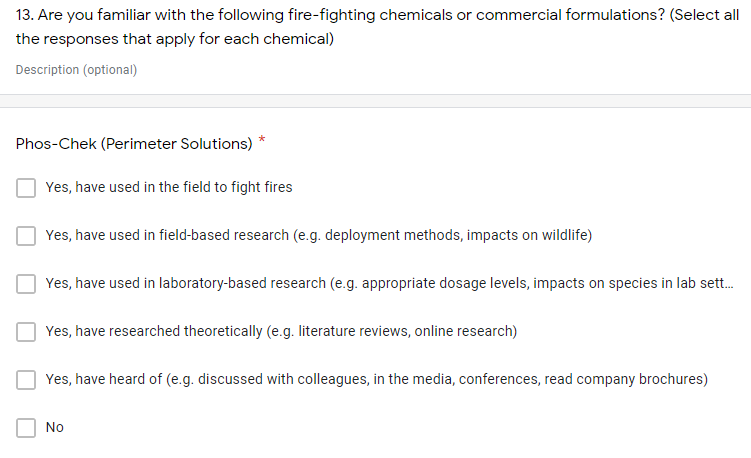


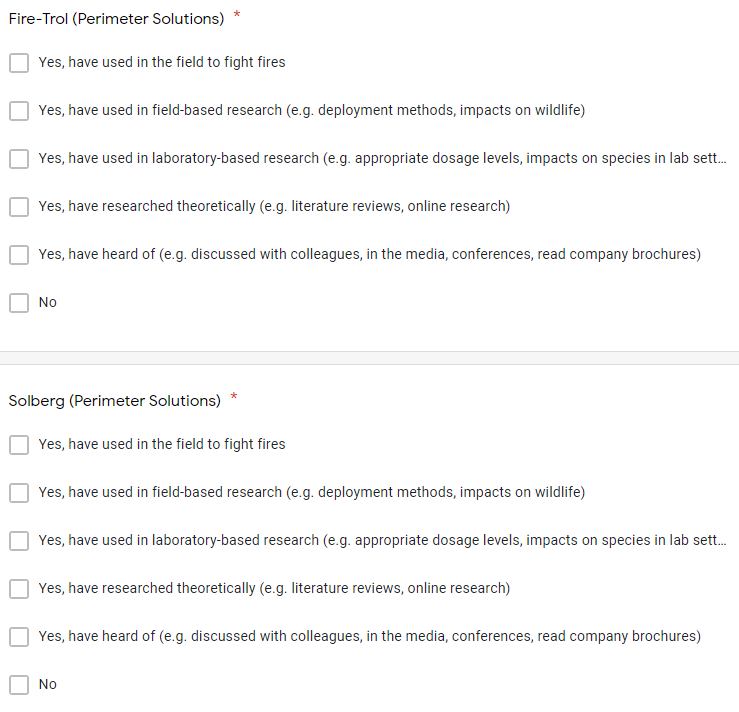

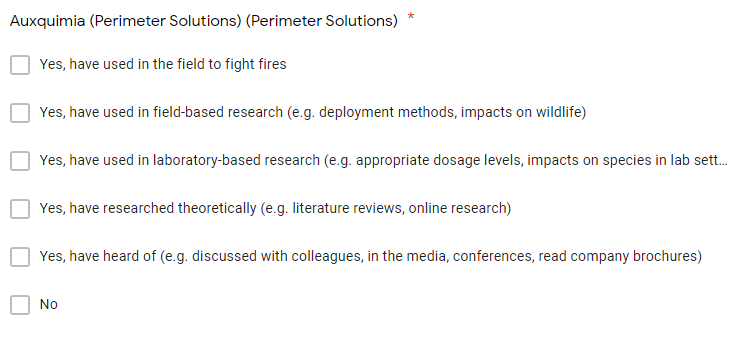


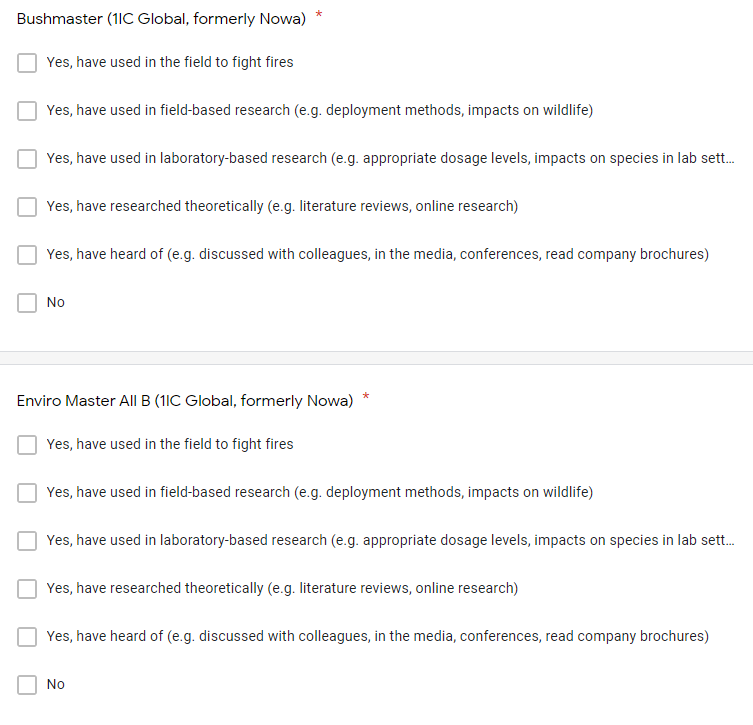

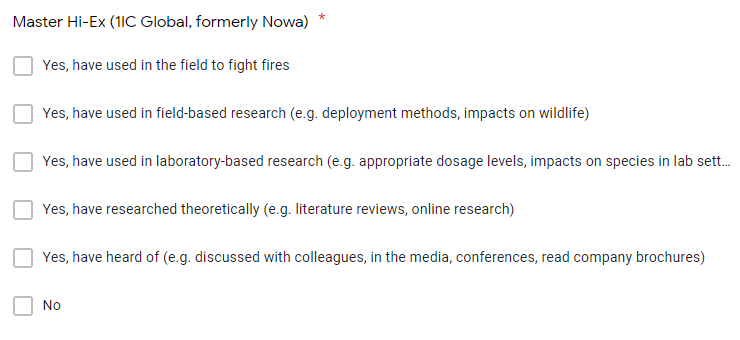


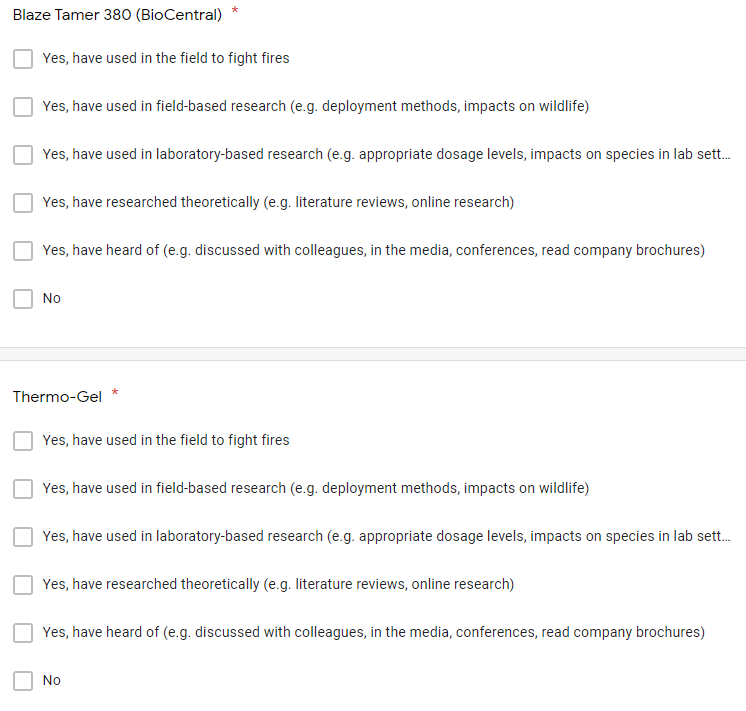

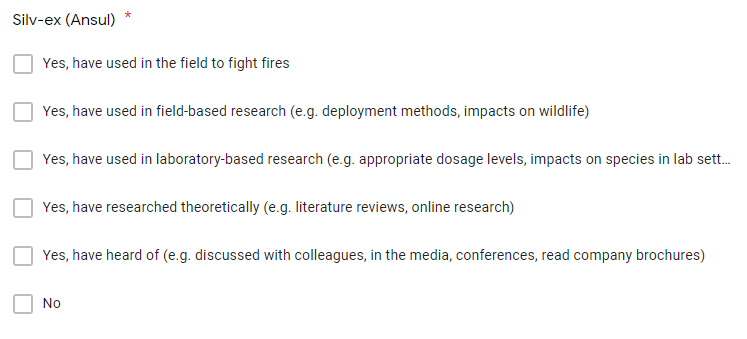


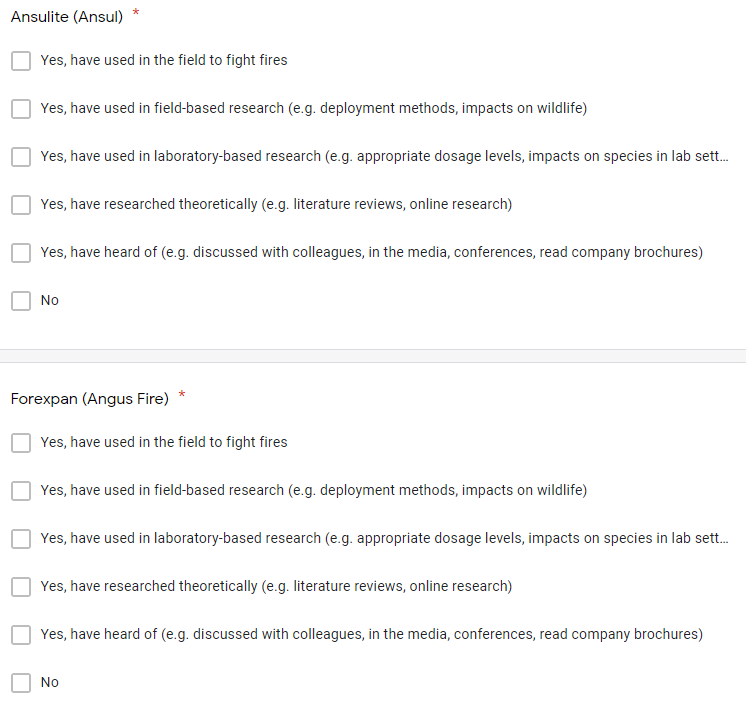


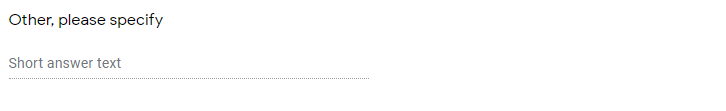


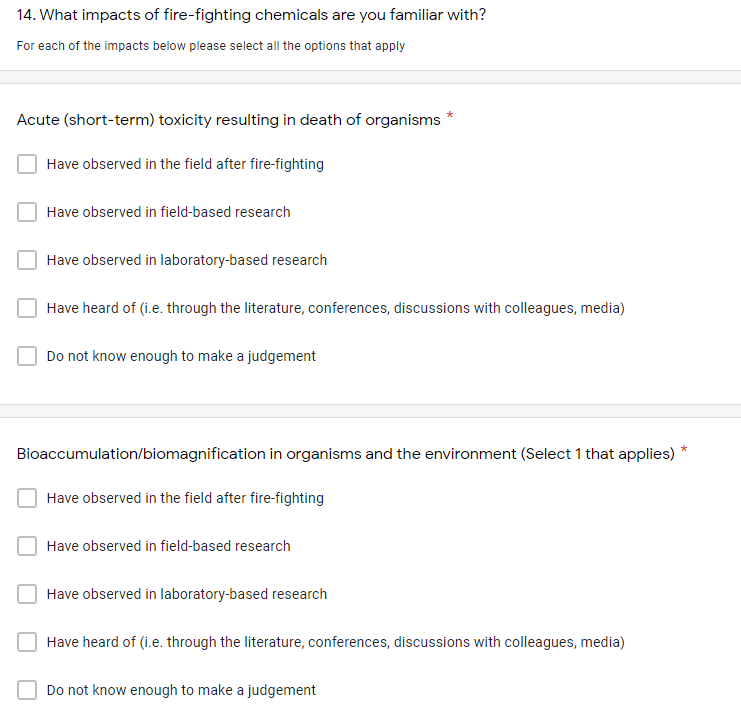


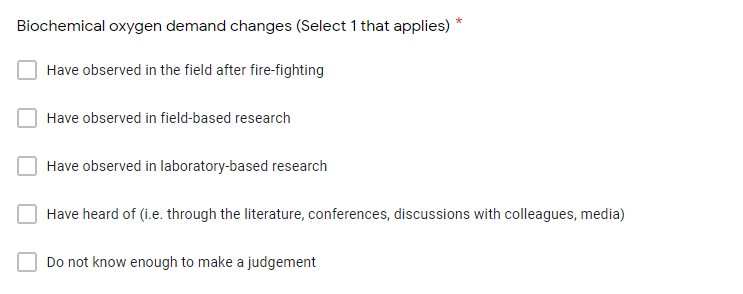


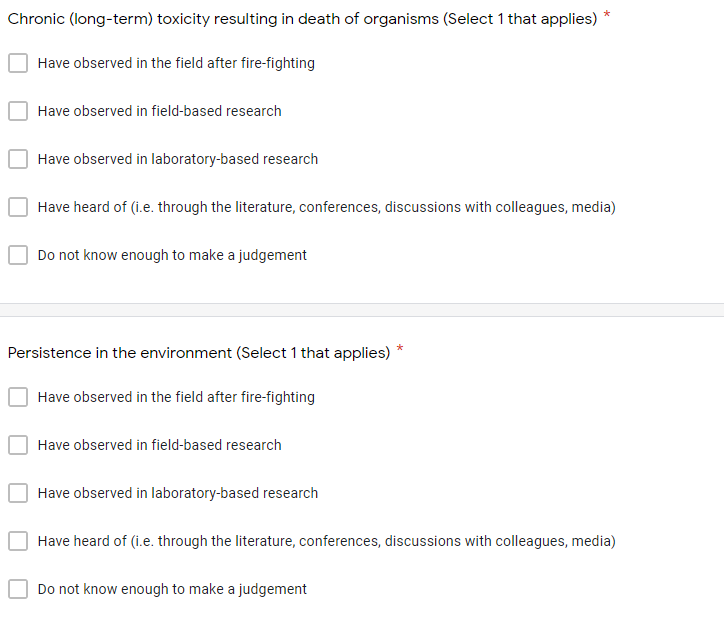


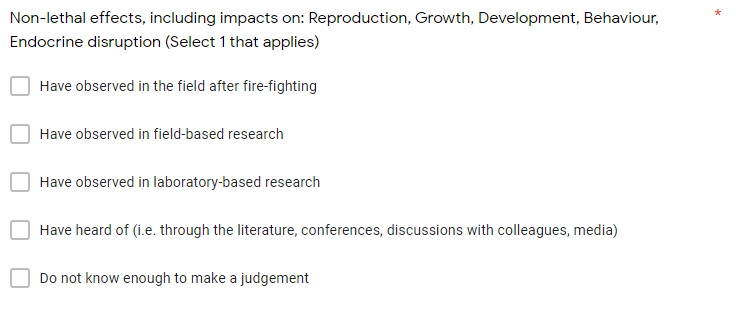


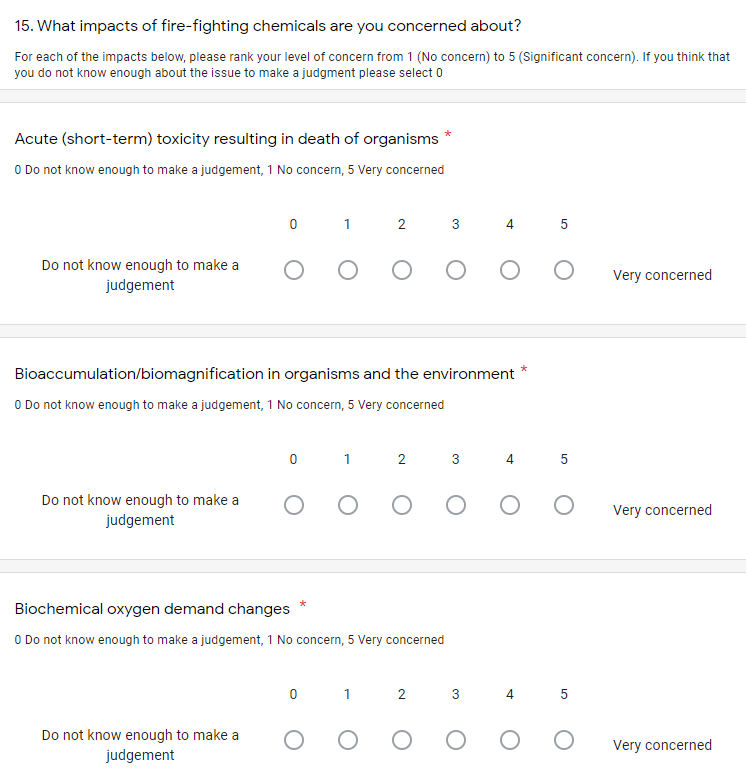


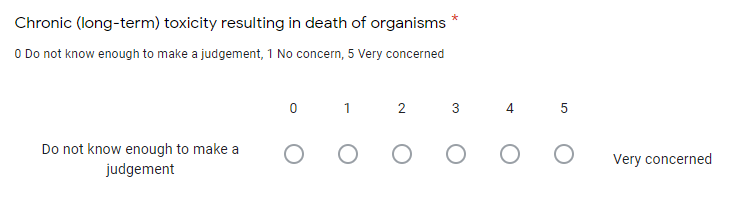


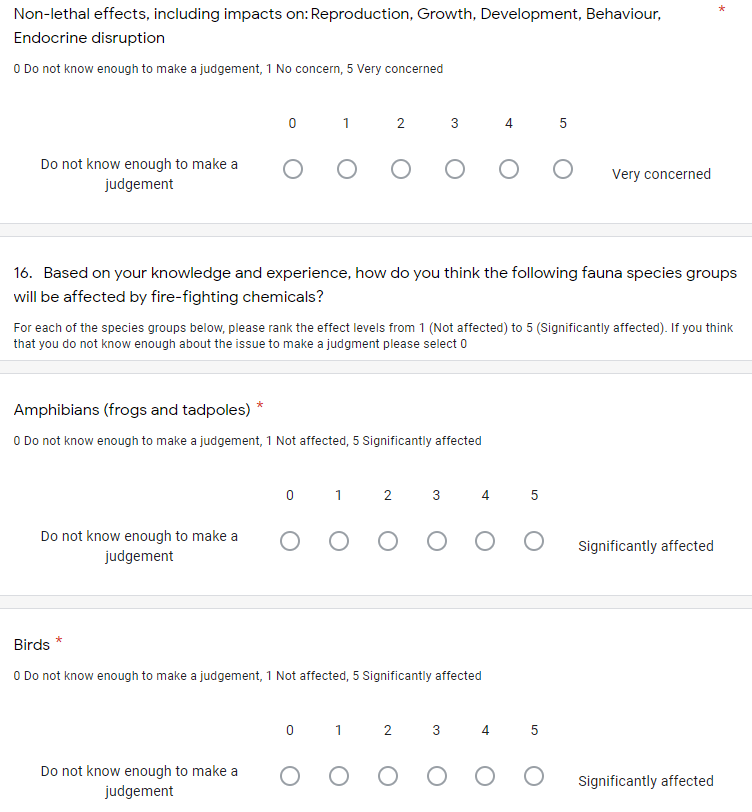


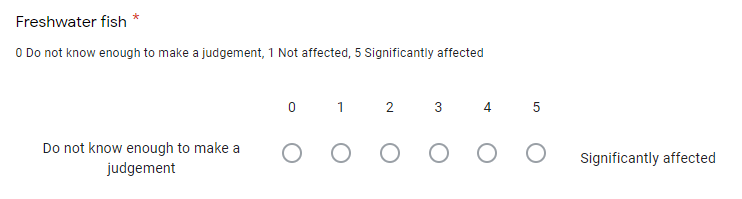


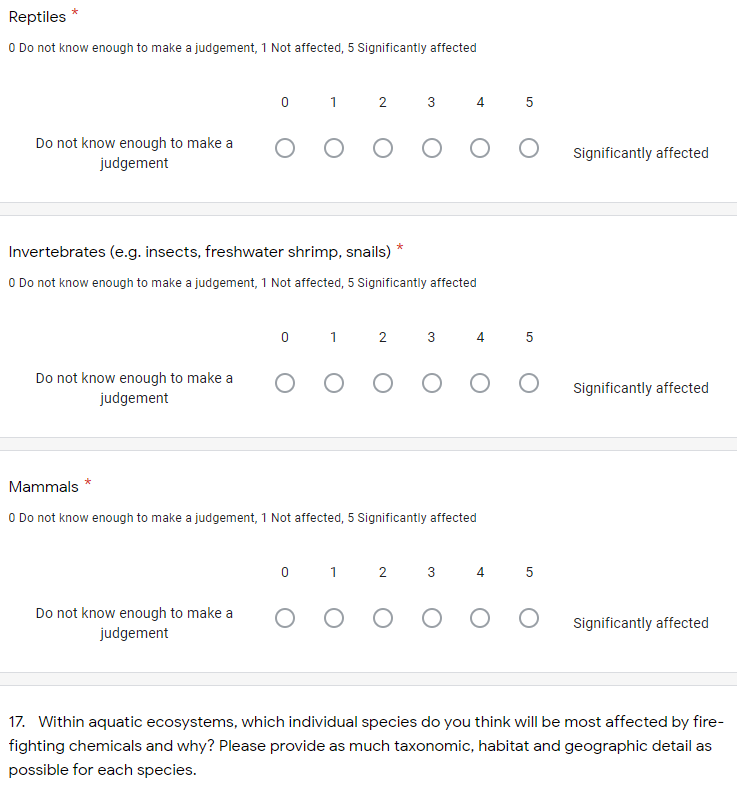

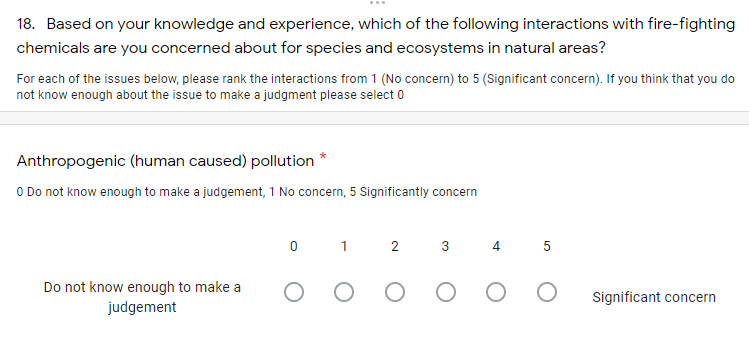


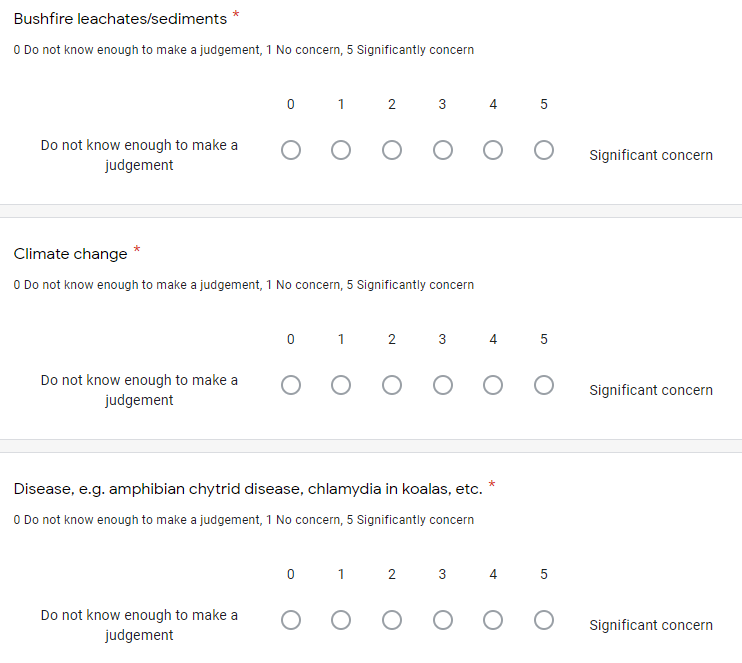

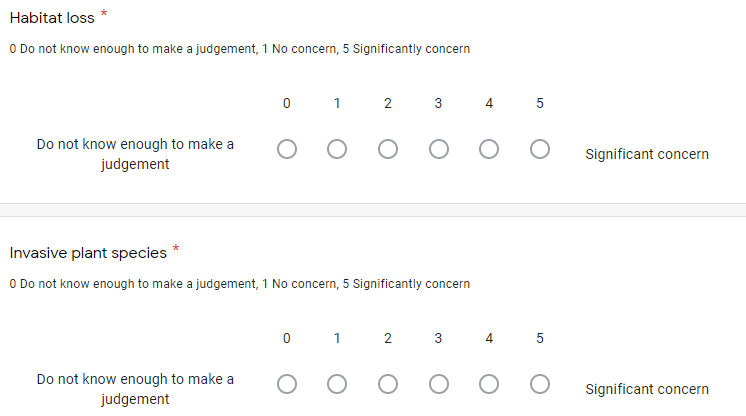


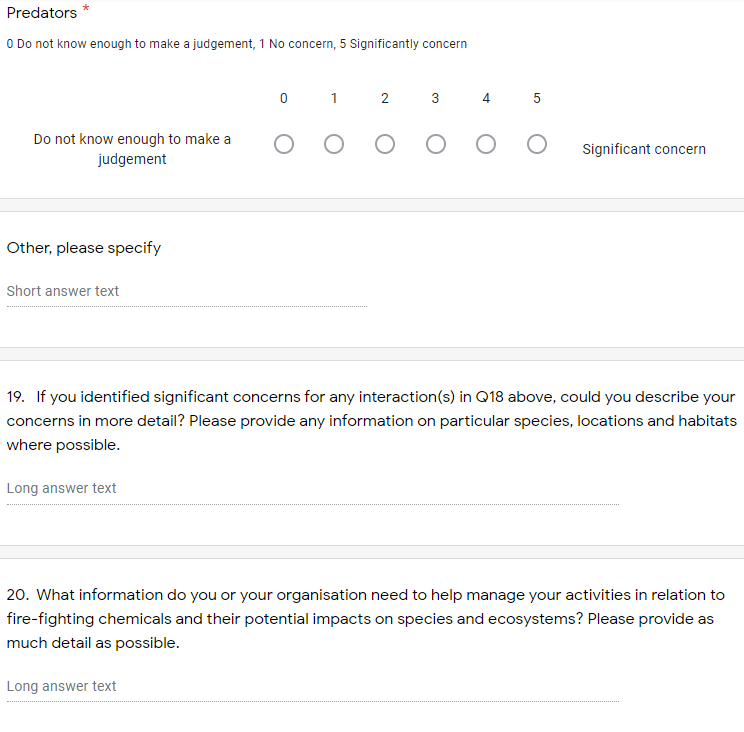

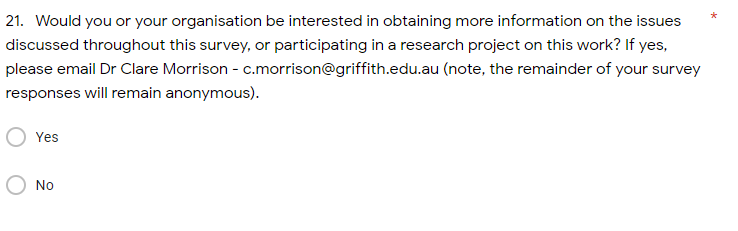


## Search terms for literature review informing the survey questionnaire

Two different search strings were used for Scopus and Web of Science, all years, peer-reviewed, English language. The first search focused on aquatic taxa, the second search on fauna more broadly to capture non-aquatic taxa. The results from all searches were combined to develop the questionnaire.

1. **Scopus**

TITLE-ABS-KEY ( frog  OR  anura*  OR  amphibia*  OR  salamander  OR  caudata  OR  newt  OR  caecilian*  OR  tadpole  OR  toad  OR  xenopus  OR  *fish*  OR  trout  OR  minnow  OR  salmon*  OR  carp  OR  *invertebrate  OR  daphni*  OR  amphipod  OR  aquatic  OR  chironomid*  OR  porifera  OR  nematod*  OR  mollusc*  OR  bivalve  OR  planaria  OR  shrimp  OR  crustacean )  AND  TITLE-ABS-KEY ( "fire fighting"  OR  firefighting  OR  ( fire  W/5  retardant )  OR  ( fire  W/5  suppress* )  OR  ( fire  W/5  foam )  OR  ( fire  W/5  foam )  OR  ( fire  W/5  chemical )  OR  ( fire  W/5  surfactant )  OR  ( foam  W/5  extinguish* )  OR  ( chemical  W/5  extinguish* )  OR  ( foam  W/5  suppress* ) )

**Web of Science**

TS=( frog  OR  anura*  OR  amphibia*  OR  salamander  OR  caudata  OR  newt  OR  caecilian*  OR  tadpole  OR  toad  OR  xenopus  OR  *fish*  OR  trout  OR  minnow  OR  salmon*  OR  carp  OR  *invertebrate  OR  daphni*  OR  amphipod  OR  aquatic  OR  chironomid*  OR  porifera  OR  nematod*  OR  mollusc*  OR  bivalve  OR  planaria  OR  shrimp  OR  crustacean )  AND TS=( "fire fighting"  OR  firefighting  OR  ( fire  W/5  retardant )  OR  ( fire  W/5  suppress* )  OR  ( fire  W/5  foam )  OR  ( fire  W/5  foam )  OR  ( fire  W/5  chemical )  OR  ( fire  W/5  surfactant )  OR  ( foam  W/5  extinguish* )  OR  ( chemical  W/5  extinguish* )  OR  ( foam  W/5  suppress* ) )

1. **Scopus**

TITLE-ABS-KEY (fauna OR animal OR mammal OR bird OR reptile)  AND  TITLE-ABS-KEY ( "fire fighting"  OR  firefighting  OR  ( fire  W/5  retardant )  OR  ( fire  W/5  suppress* )  OR  ( fire  W/5  foam )  OR  ( fire  W/5  foam )  OR  ( fire  W/5  chemical )  OR  ( fire  W/5  surfactant )  OR  ( foam  W/5  extinguish* )  OR  ( chemical  W/5  extinguish* )  OR  ( foam  W/5  suppress* ) )

**Web of Science**

TS=(fauna OR animal OR mammal OR bird OR reptile)  AND TS=( "firefighting"  OR  firefighting  OR  ( fire  W/5  retardant )  OR  ( fire  W/5  suppress* )  OR  ( fire  W/5  foam )  OR  ( fire  W/5  foam )  OR  ( fire  W/5  chemical )  OR  ( fire  W/5  surfactant )  OR  ( foam  W/5  extinguish* )  OR  ( chemical  W/5  extinguish* )  OR  ( foam  W/5  suppress* ) )

## Expert demographics

**Table S1.** Summary of participant demographics

| **Characteristic** | **Category** | **N (%)** |
| --- | --- | --- |
| *State/Territory* | Australian Capital Territory | 5 (11%) |
|  | New South Wales | 5 (11%) |
|  | Northern Territory | 2 (4%) |
|  | Queensland | 17 (37%) |
|  | South Australia | 3 (6%) |
|  | Tasmania | 0 (0%) |
|  | Victoria | 10 (22%) |
|  | Western Australia | 4 (9%) |
| *Organisation type* | Academic | 11 (24%) |
|  | Fire service (national, state, rural) | 6 (13%) |
|  | Government (local, state, Federal) | 22 (48%) |
|  | NGO | 2 (4%) |
|  | Private company (e.g., business, zoo, consultant) | 5 (11%) |
| *Primary role* | Research - academic | 18 (39%) |
|  | Conservation or land management | 15 (33%) |
|  | Policy development or implementation | 4 (9%) |
|  | Fire management or fighting | 7 (15%) |
|  | Disaster management | 2 (4%) |
| *Length of time in role* | < 1 year | 0 (0%) |
|  | 1-5 years | 10 (22%) |
|  | 5-10 years | 3 (6%) |
|  | 10-20 years | 15 (33%) |
|  | 20+ years | 18 (39%) |
| *Highest level of education* | Secondary school | 0 (0%) |
|  | Technical education | 5 (11%) |
|  | University undergraduate degree | 12 (26%) |
|  | University postgraduate degree | 29 (63%) |
| *Direct experience with fire management* | Yes | 33 (72%) |
|  | No | 13 (28%) |
| *Direct experience with fire impacts* | Yes | 35 (76%) |
|  | No | 11 (24%) |

## Supplementary Figures


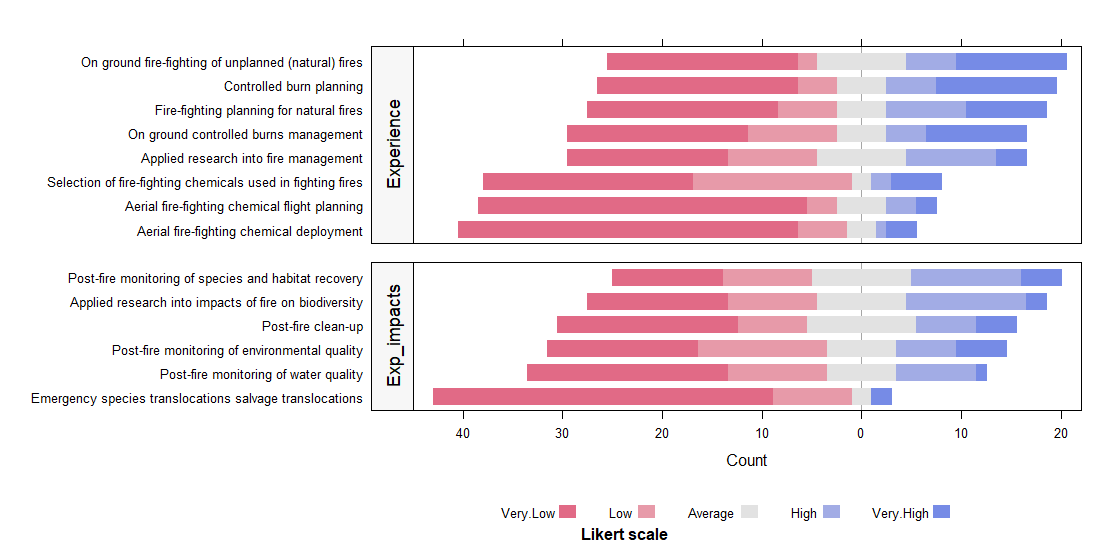


**Figure S1**. Respondents experience with different aspects of fire management (top), and respondent’s direct experience with impacts of fires in natural areas (bottom)
